# Association Between Genetic Risks for Obesity and Working Memory in Children

**DOI:** 10.3389/fnins.2021.749230

**Published:** 2021-09-22

**Authors:** Nagahide Takahashi, Tomoko Nishimura, Taeko Harada, Akemi Okumura, Toshiki Iwabuchi, Md. Shafiur Rahman, Hitoshi Kuwabara, Shu Takagai, Yoko Nomura, Nori Takei, Kenji J. Tsuchiya

**Affiliations:** ^1^Department of Child and Adolescent Psychiatry, Nagoya University Graduate School of Medicine, Nagoya, Japan; ^2^Research Center for Child Mental Development, Hamamatsu University School of Medicine, Hamamatsu, Japan; ^3^United Graduate School of Child Development, Hamamatsu University School of Medicine, Hamamatsu, Japan; ^4^Department of Psychiatry, Hamamatsu University School of Medicine, Hamamatsu, Japan; ^5^Department of Child and Adolescent Psychiatry, Hamamatsu University School of Medicine, Hamamatsu, Japan; ^6^Queens College and Graduate Center, City University of New York, New York, NY, United States

**Keywords:** polygenic risk score, obesity, cognition, GWAS, child development

## Abstract

**Introduction:** Obesity is highly heritable, and recent evidence demonstrates that obesity is associated with cognitive deficits, specifically working memory. However, the relationship between genetic risks for obesity and working memory is not clear. In addition, whether the effect of these genetic risks on working memory in children is mediated by increased body mass index (BMI) has not been elucidated.

**Methods:** In order to test whether the polygenic risk score (PRS) for obesity in adulthood (adulthood-BMI-PRS) is associated with working memory at 8 years of age, and whether the effect is mediated by childhood BMI, in children from the general population, participants in the Hamamatsu Birth Cohort for Mothers and Children (HBC) study in Hamamatsu, Japan, underwent testing for association of adulthood-BMI-PRS with working memory. HBC data collection began in December 2007 and is ongoing. Adulthood-BMI-PRS values were generated using summary data from the recent genome-wide association study (GWAS) undertaken in Japan, and the significance of thresholds was calculated for each outcome. Outcomes measured included the working memory index (WMI) of Weschler Intelligence Scale-4 (WISC-IV) scores and the BMI at 8 years of age. Gene-set enrichment analysis was conducted to clarify the molecular basis common to adulthood-BMI and childhood-WMI. Mediation analysis was performed to assess whether childhood-BMI of children mediated the association between adulthood-BMI-PRS and working memory.

**Results:** A total of 734 participants (377 males, 357 females) were analyzed. Adulthood-BMI-PRS was associated with lower childhood-WMI (β[SE], −1.807 [0.668]; *p* = 0.010, corrected) of WISC-IV. Gene-set enrichment analyses found that regulation of neurotrophin Trk receptor signaling (β[SE], −2.020 [6.39]; *p* = 0.002, corrected), negative regulation of GTPase activity (β[SE], 2.001 [0.630]; *p* = 0.002, corrected), and regulation of gene expression epigenetic (β[SE], −2.119 [0.664]; *p* = 0.002, corrected) were enriched in BMI in adulthood and WMI in childhood. Mediation analysis showed that there is no mediation effect of childhood-BMI between the adulthood-BMI-PRS and working memory deficits in children.

**Conclusion:** Adulthood-BMI-PRS was associated with working memory among children in the general population. These genetic risks were not mediated by the childhood-BMI itself and were directly associated with working memory deficits.

## Introduction

Accumulating evidence shows that obesity is associated with deficits in neurocognitive functioning, such as deficits in working memory in children (Smith et al., 2011; Cornier et al., 2013; Crone and Steinbeis, 2017). Obesity is associated with adiposity, cellular stress, and excessive inflammation, all of which can lead to insulin resistance and cerebral structural alteration (Unamuno et al., 2018). Numerous neuroimaging studies have found structural alterations in obese children (Maayan et al., 2011; Veit et al., 2014; Sharkey et al., 2015; Saute et al., 2018); however, due to the limited sample size and methodological difference, results regarding the relationship between obesity, cortical thickness, and cognitive deficits have been inconsistent (Maayan et al., 2011; Sharkey et al., 2015; Saute et al., 2018). A recent large neuroimaging study reported that higher body mass index (BMI) was associated with lower working memory, and this association was mediated by reduced prefrontal cortex thickness in children (Laurent et al., 2019).

Although obesity is a complex phenotype, the role of genetic factors in the development of obesity remains undisputed. Additionally, a recent genome-wide association study (GWAS) identified 85 loci associated with BMI in adults from the Japanese general population (Akiyama et al., 2017). Furthermore, a previous study showed that BMI was genetically correlated with general cognitive function in adults using LD score regression analysis (*r*_*g*_ = 0.51) (Marioni et al., 2016). However, Riggs et al. found that alteration in working memory is antecedent to weight gain in children, suggesting the possibility that cortical thickness or subsequent cognitive dysfunctions cause obesity (Riggs et al., 2010; Groppe and Elsner, 2017). Together, it is reasonable to hypothesize that children with genetic risks for obesity might have a higher risk for neurocognitive deficits compared to those with low genetic risks for obesity and the genetic risks for obesity potentially directly affect working memory without mediation of BMI.

As such, we examined (1) whether polygenic risk scores (PRSs) for BMI in adulthood (adulthood-BMI-PRS) are associated with deficits in working memory, leveraged on our birth cohort, composed of representative samples of Japanese, and (2) whether childhood-BMI mediated the association between adulthood-BMI-PRS and working memory.

## Materials and Methods

### Participants

Participants included infants (*n* = 832; 426 boys, 406 girls) born in Japan between December 2007 and June 2011. The recruitment procedures are described in detail in our previous study (Takagai et al., 2016). The study procedures were approved by the ethical committee. Written informed consent was obtained from each mother for the participation of her infant. Participants with parents of non-Japanese descent were excluded from the study (*n* = 8). No other screening, such as neurodevelopmental disorders or psychiatric disorders was conducted for the analysis. Hamamatsu University School of Medicine and the University Hospital Ethics Committee accepted the study methods.

### Measurement

Working memory was assessed, using the working memory index (WMI) of the Wechsler Intelligence Scale for Children-4 (WISC-IV) when the children became 8 years of age. Information pertaining to BMI was obtained on the same day when the WISC-IV assessments were carried out.

### Genotyping, Quality Control, and Imputation

Genotyping was conducted using the Japonica array designed specifically for single-nucleotide polymorphism (SNP) genotyping for a Japanese population (Kawai et al., 2015). The quality controls retaining SNPs and subjects were as follows: missing data for SNP < 0.02, Pi-hat calculated by identity-by-descent analysis < 0.2, SNP Hardy-Weinberg equilibrium of *p* > 10^–6^, and minor allele frequency > 0.01. Genotyping imputation was performed using BEAGLE 5.0 (Browning et al., 2018) to the Japanese reference panel phase 3 of 1000 Genome Project. SNPs with an imputation INFO score < 0.8 were excluded. We also excluded SNPs located within the MHC region, because of high linkage disequilibrium (LD) in this region. The number of SNPs analyzed for PRS was 5,606,655.

## Statistical Analysis

### Polygenic Risk Score Analysis

PRS was generated by PRSice-2 (Choi and O’Reilly, 2019) using a recent BMI-GWAS in the Japanese adult population as a discovery cohort^1^ (Akiyama et al., 2017). Four main components calculated with PLINK 1.9 (Chang et al., 2015) were used to account for population stratification. The criterion for SNP clumping was pairwise linkage disequilibrium of *r*^2^< 0.1 within a 1-Mb window. PRSs were calculated with different *p*-value thresholds: 0.05, 0.1, 0.2, 0.3, 0.4, and 0.5. Standardized PRS scores (mean = 0; standard deviation = 1) were used for the analyses. *p*-values for WMI were corrected using 10,000 permutation tests. Sex and small-for-gestational-age (SGA) were included as covariates. The statistical power of the PRS at each *p*-value threshold was calculated using the AVENGEME R-package (Dudbridge et al., 2018). Since SNP-based heritability (h^2^ SNP) estimated from the original BMI study was 0.29812, all generated PRSs showed adequate power between 90 and 100%.

Gene-set enrichment analyses were conducted using PRSet (Choi et al., 2021) to identify gene sets that contain SNPs associated with both BMI in adulthood and WMI in childhood. Gene ontology (GO) sets (c5: biological process) were obtained from the MSigDB database^2^ and used for the analyses by PRSice-2 (Choi and O’Reilly, 2019). The *p*-value threshold for PRSet was set at 1, since gene-set PRSs containing a small portion of SNPs may be unrepresentative of the entire gene set (Fanelli et al., 2020). The *p*-values for PRSet were corrected by 10,000 permutation tests.

### Mediation Analysis

Mediation analysis was performed to assess whether childhood-BMI mediated the association between adulthood-BMI-PRS and working memory. Best-fit PRSs computed with highest *R*^2^, obtained from linear regression analyses, were used for the medication analysis. The R package “lavaan” was used, and the significance of indirect effect of childhood-BMI was assessed by 1,000 bootstrap at a 95% confidential interval (Rosseel, 2012). The data were tested for normal distribution by a Shapiro–Wilk test.

## Results

### Association Between Adulthood-Body Mass Index-Polygenic Risk Score and Childhood-Working Memory Index

Participant characteristics are summarized in Table 1. Genotyping quality control and identity-by-decent analysis were used to remove 98 individuals from the analysis, resulting in a total of 726 participants (373 males, 353 females) for further analysis. The adulthood-BMI-PRS was significantly associated with lower WMI of WISC-IV at various *p*-value thresholds (Table 2).

**TABLE 1:** Sample characteristics: participating children and their parents.

	Mean (*SD*)
Birthweight (g)	2935.1 (444.3)
Gestational age at birth (weeks)	38.9 (1.6)
Paternal age at birth (years)	33.5 (5.7)
Maternal age at birth (years)	29.3(5.2)
Household income (million JPY)	6.1 (2.7)
BMI	16.2 (2.4)
Gender	*n* (%)
Male	439 (50.1)
Female	437 (49.9)
**Ethnicity**	
Japanese	868
Mixed (Caucasian)	5
Mixed (Latino)	3
**Small for gestational age**	
<10th percentile	785 (89.6)
10th–100th percentile	91 (10.4)
**Placenta-to-birthweight ratio (twin excluded)**	
<10th percentile	164 (18.7)
10th–100th percentile	712 (81.3)
**Paternal education**	
<12 years	65 (7.4)
12years and longer	811 (92.6)
**Maternal education**	
<12 years	38 (4.3)

*SD, standard deviation; BMI, body mass index.*

**TABLE 2:** Association between BMI PRS and WMI of WISC-IV.

WISC items	*p*-value threshold	*R* ^2^	β	SE	*p*-values*
WMI	0.05	0.023	−1.416	0.675	0.129
	0.1	0.024	−1.470	0.687	0.099
	0.2	0.028	−1.713	0.674	**0.030**
	0.3	0.030	−1.807	0.668	**0.010**
	0.4	0.028	−1.691	0.664	**0.020**
	0.5	0.027	−1.616	0.667	**0.040**

*BMI, body mass index; PRS, polygenic risk score; WISC-IV, Wechsler Intelligence Scale for Children-4; SNP, single-nucleotide polymorphism; WMI, working memory index.*

*The number of SNPs used to calculate PRSs were 22,093 (p < 0.05), 33,299 (p < 0.1), 50,147 (p < 0.2), 63,106 (p < 0.3), 73,554 (p < 0.4), and 82,278 (p < 0.5).*

*p-values were corrected for 10,000 permutation tests. Statistically significant p-values were shown in bold.

### Gene-Set Enrichment Analysis of Adulthood-Body Mass Index-Polygenic Risk Score and Childhood-Working Memory Index

Gene-set enrichment analysis identified that several gene ontologies, such as regulation of neurotrophin Trk receptor signaling (β[SE], −2.020 [6.39]; *p* = 0.002, corrected), negative regulation of GTPase activity (β[SE], 2.001 [0.630]; *p* = 0.002, corrected), and regulation of gene expression epigenetic (β[SE], −2.119 [0.664]; *p* = 0.002, corrected), were enriched in BMI in adulthood and WMI in children (Table 3).

**TABLE 3:** Top 20 gene sets significantly enriched for WMI and BMI.

Gene-sets	*R* ^2^	β	SE	Number of SNP	*p*-value
Regulation of neurotrophin Trk receptor signaling pathway	0.036	−2.020	0.639	99	0.002
Negative regulation of GTPase activity	0.036	2.001	0.630	320	0.002
Regulation of gene expression epigenetic	0.036	−2.119	0.664	1,360	0.002
DNA synthesis involved in DNA repair	0.036	2.029	0.636	221	0.003
Histone H3 K9 demethylation	0.032	1.893	0.658	105	0.004
Glutathione derivative biosynthetic process	0.031	−1.802	0.639	61	0.005
Negative regulation of bmp signaling pathway	0.033	−1.914	0.650	241	0.005
Positive regulation of neuron death	0.031	−1.914	0.681	400	0.006
Nucleoside diphosphate biosynthetic process	0.030	−1.905	0.694	45	0.006
Oligodendrocyte progenitor proliferation	0.030	−1.709	0.634	60	0.006
Layer formation in cerebral cortex	0.030	−1.788	0.662	92	0.007
Endoplasmic reticulum mannose trimming	0.029	1.711	0.643	84	0.008
Positive regulation of tor signaling	0.030	−1.822	0.661	172	0.008
Negative regulation of histone methylation	0.029	1.703	0.647	146	0.010
Canonical wnt signaling pathway	0.029	1.819	0.680	1,811	0.011
Negative regulation of RNA metabolic process	0.029	−1.723	0.649	171	0.011
Cellular response to brain derived neurotrophic factor stimulus	0.027	−1.717	0.700	62	0.015
Lateral ventricle development	0.027	1.631	0.667	96	0.016
Regulation of snare complex assembly	0.027	−1.576	0.650	62	0.016
Postsynaptic cytoskeleton organization	0.026	1.563	0.652	55	0.017

*WMI, working memory index; BMI, body mass index; SNP, single-nucleotide polymorphism; SE, standard error.*

*p-values were corrected for 10,000 permutation tests.*

### Mediation Analysis of Adulthood- Body Mass Index-Polygenic Risk Score, Childhood-Body Mass Index, and Childhood-Working Memory Index

**FIGURE 1:**
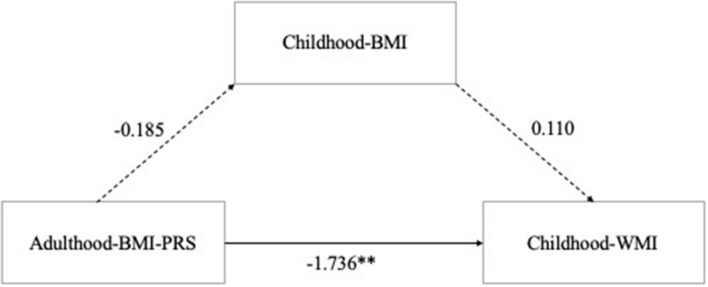
Mediation analysis of Adulthood-BMI-PRS, Childhood-BMI, and Childhood-Working memory. The solid line indicates the path that was statistically significant and the dashed lines indicate those that were estimated but not statistically significant. BMI, body mass index; PRS, polygenic risk score. ^∗∗^*p* < 0.01.

All data including adulthood-BMI-PRS, childhood-BMI, and childhood-WMI were normally distributed by a Shapiro–Wilk test (*p* > 0.05). Mediation analysis was conducted for BMI-PRS (best fit *p*-value threshold at 0.319), childhood-BMI, and childhood-WMI. Analysis revealed that although there was a significant total effect of BMI-PRS on WMI (β[SE], −1.874 [0.633]; *p* = 0.004), no indirect effect of childhood-BMI (β[SE], 0.110 [0.102]; *p* = .280) was observed, indicating that most of the effect originated from the direct effect of BMI-PRS (β[SE], −1.736 [0.638]; *p* = 0.006) (Figure 1). Furthermore, regression analysis showed that there is no association between adulthood-BMI-PRS and childhood-BMI in the present study (β[SE], −0.185 [0.130]; *p* = 0.156).

## Discussion

We report that genetic risks for obesity are linked to working memory deficits in children. This finding partially supports the previous report (Laurent et al., 2019); however, we found that the effect of BMI-PRS on WMI was not mediated by childhood-BMI, indicating that childhood-BMI itself does not affect this cognitive domain directly, but the genes involved in adulthood-BMI are responsible for these functions in the brain. This finding is also consistent with a previous study showing strong genetic correlation between BMI and general cognitive functions in adults using LD score regression analysis (Marioni et al., 2016).

Multiple reasons could be considered for the lack of association between adulthood-BMI-PRS and childhood-BMI itself in our study population. First, as we targeted 8-year-old children, it might be too early to detect the effect of genes related to adulthood-BMI on BMI in children. Second, there is a possibility that genes related to adulthood-BMI are different between children and adults.

In accordance with the previous GWAS results (Akiyama et al., 2017), GO sets commonly enriched in adulthood-BMI and childhood-WMI have been reported to be involved in the brain cortical maturation (Anton-Fernandez et al., 2015; Budday et al., 2015; Lian and Sheen, 2015). For example, genes in oligodendrocyte progenitor proliferation or layer formation in cerebral cortex have been reported to be expressed in the brain from the early postnatal period.^3^ Among these GOs, it is noteworthy that regulation of gene expression epigenetic was identified, since a recent GWAS demonstrated that a SNP on the HDAC4 gene was associated with selective attention (Pinar et al., 2018). Taken together, it can be considered that these genes are involved in brain maturation and working memory directly, without mediation of body weight.

## Limitations

There are a few limitations in our study. First, compared to the previous study (Laurent et al., 2019), the average childhood-BMI [mean (SD), 18.64 (3.9) in the previous study vs. 16.2 (2.4) in this study] was lower in our cohort; thus, there is a possibility that we did not target a population with a broad range of BMI and, thus, could not detect an effect of adulthood-BMI-PRS on childhood-BMI in this study. Incidence of children classified as overweight (i.e., 85–95%) in this study was 9.68%, compared to 13.4% in the previous study. Similarly, the incidence of children classified as obese (i.e., > 95%) was 6.98% in this study, compared to 15.4% in the previous study (Laurent et al., 2019). Second, as BMI does not always reflect obesity, more sophisticated measurement to evaluate obesity, such as body fat percentage, is needed (Nickerson et al., 2018).

## Conclusion

In this study, the adulthood-BMI-PRS was associated with working memory among children in the general population. These genetic risks were directly associated with working memory deficits, and not mediated by children’s BMI. Future studies are warranted in order to replicate these findings.

## Data Availability Statement

The data generated for this study is subject to the following licenses/restrictions: Privacy and Confidentiality of Participants. Requests to access these datasets should be directed to KT, tsuchiya@hama-med.ac.jp.

## Ethics Statement

The studies involving human participants were reviewed and approved by Hamamatsu University School of Medicine and the University Hospital Ethics Committee. Written informed consent to participate in this study was provided by the participants’ legal guardian/next of kin.

## Author Contributions

NaT had full access to all the data used in the study and takes responsibility for the integrity of the data and accuracy of the data analysis, and statistical analysis. NaT, NoT, and KT study concept and design. NaT and KT drafting the manuscript. NoT and KT study supervision. TH, TN, and AO administrative, technical, and material support. All authors contributed significantly to the study and the creation of this manuscript, acquisition, analysis, interpretation of data, and critical revision of the manuscript for important intellectual content.

## Conflict of Interest

The authors declare that the research was conducted in the absence of any commercial or financial relationships that could be construed as a potential conflict of interest.

## Publisher’s Note

All claims expressed in this article are solely those of the authors and do not necessarily represent those of their affiliated organizations, or those of the publisher, the editors and the reviewers. Any product that may be evaluated in this article, or claim that may be made by its manufacturer, is not guaranteed or endorsed by the publisher.

## Footnotes

1
https://humandbs.biosciencedbc.jp/hum0014-v23

2
https://www.gsea-msigdb.org/gsea/msigdb/index.jsp

3
informatics.jax.org
